# The Present-Day Tendencies of Dental Practice*Read before the Maryland State Dental Association, Baltimore, Md., June 22, 1920. Reprinted from *Dental Cosmos*, November, 1920.

**Published:** 1921-01

**Authors:** L. Pierce Anthony


### THE PRESENT-DAY TENDENCIES OF DENTAL PRACTICE*

BY L. PIERCE ANTHONY, D.D.S.

The world-wide spirit of unrest and discontent as the resultant after-math of the recent war, or as one writer has termed it, the moral shell shock from which the social world is suffering, has very perceptibly affected the world of dentistry and has been expressed in a demand for a general dental house-cleaning. The reaction of the dental profession has been a forcible realization of the necessity of the adoption of methods of practice which will more nearly meet the increasing demands upon dentistry, and the most hopeful direction seems to be that of prevention.

We have for many decades been endeavoring to impress upon the medical profession and the public the importance of the oral cavity as a factor in bodily health and disease and to convince them of the latent potentialities for evil that the oral cavity harbors. The mouth cavity has at last received full recognition as an important, if not *the most* important, organ of the body in the life process and in many cases its finality—death. It was not, however, until the radiograph came to be generally applied to dental practice that such recognition came, and the impetus it has received, through the medical profession and the public, was so overwhelming that at one time it threatened calamity to the dental profession. Indeed, that time is so recent that we have not yet entirely recovered our equilibrium.

### RADICALISM

From absolute disregard of the human mouth as a factor in bodily disease to considering it the most potent

* Read before the Maryland State Dental Association, Baltimore, Md., June 22, 1920. Reprinted from *Dental Cosmos*, November, 1920.

factor in the causation of all bodily ills would seem to be a giant's stride, but it is one which the medical profession can and did make with greatest ease. In fact, the medical profession has been notoriously facile in its chameleon-like changes from one fad to another for many centuries. It is prolific in fads which soon pass into the oblivion they deserve, and herein is the hope of the future in our present newly-developed relationship to our mother profession. The medical profession, we feel, is unquestionably equal to the task of retracing its extreme radical steps toward the elimination of all diseased dental conditions to a safe and sane ground. Indeed we are happy to see that some of the leading medical men have already seen the dangerous extreme toward which they were tending and have called a halt, and are now in a mood to concede the claims of the more conservative element of dentistry. This is as it should be, for if the focal oral infection tide had continued to swell we should soon have become a toothless race. The physiologic function of the mouth and teeth as the first and one of the most important of the organs taking part in the process of digestion seemed to have been entirely disregarded and our path of vision to the ideal in dentistry seemed hopelessly obscured.

The opprobrium of such radicalism is not all upon the medical profession; some members of the dental profession have gone to as great extremes as the medical profession and some are still exhibiting hysterical symptoms of radicalism, one prominent English dentist having gone to the absurd extreme of extracting all of the teeth of one of his young children in the hope of avoiding all the numerous evils which have been attributed to focal infection in the oral cavity.

Thus we see that the tendency to faddism is as pronounced in the dental profession as in the medical, and as long ago as 1882 the well-known Dr. Welch, then the editor of *Items of Interest*, in commenting on this tendency likened the dental profession to Barnum's elephant on a see-saw: "His anxiety to get to the extreme height of the plank makes him pass the center, when he finds himself suddenly brought to the ground. Though he often repeats his experiment he is seldom content with the most elevated position attainable—the center." However, 'tis an ill wind that blows no good, and the beneficial result of our most recent fetish has been to awaken the dental profession to the sins of commission and omission in the past and to arouse in all ranks the desire to improve the methods of practice so that we may creditably perform the mission of dentistry, namely, to save teeth rather than extract them.

### THE RADIOGRAPH

Much of the radicalism above referred to has come from a misinterpretation of radiographic findings, and this valuable addition to our practice will eventually, we feel, prove to be one of the greatest advances made in many decades past. When we have arrived at the point where we can properly interpret our findings, we do not believe they will point, as some seem to think at present, inevitably to the removal of every affected tooth. Too much reliance is being placed upon the X-ray film and its interpretation as the final diagnosis of all cases. It will eventually prove an invaluable adjunct to dental practice, but it will be as a diagnostic aid to clinical manifestations.

The medical profession has challenged us to make the next greatest step in preventive medicine; we are taking that step and the public is impatiently awaiting the result. We can not make the step successfully though, without the co-operative aid of the medical profession. Mayo says the trend of modern medicine and dentistry is bringing their fields more closely together. The recent war was undoubtedly a means to this desirable end, but more recently this co-operation in some quarters has been more apparent than real.

### CROWN AND BRIDGE WORK

The present system, or rather lack of system, in the practice of crown and bridge work seems to have received more censure than any other phase of practice, but we believe the greatest fault lies in treatment and filling of root canals, crown and bridge work being only the avenue of expression of the evils of faulty root canal work, and when we have arrived at a definitely standardized and reliable procedure for filling root canals, bridge work will become a safe and reliable means of restoration.

Efforts individual and collective have been exerted toward the solution of many of our problems and, while the results have not been all that we could wish, much progress is being recorded. Research work is being conducted under the auspices of the National Dental Association, but in the anxiety to produce immediate results much confusion has arisen and much effort has been misdirected. It seems that the most valuable research work that has been done in dentistry has been in the individual efforts of men qualified for such work and possessing the scientific sense—if we may so term it.

### ROOT CANAL WORK

The root canal problem, the *bête noir* of dental practice, is being attacked from many angles with considerable success, and the profession has realized that success in this field involves the same factors as in almost all phases of dentistry, namely, highly-skilled individual technique aided by strict surgical aseptic procedure. Many ingenious methods and procedures have been devised to obviate the necessity of pulp devitalization, in the form of inlays, clasps, etc., as attachments for bridge work, but at best this is merely temporizing with the situation in an effort to postpone the evil day when more of the teeth will be lost.

The cumulative result of our experiences and observations in the past, however, is that our problem can only be rationally solved through the means of prevention—prevention of caries and its consequent chain of evils through intelligently applied prophylactic measures. And to this end the profession is rapidly coming to a realization of the great assistance that may be derived from the intelligent aid of the dental hygienist. The latter will enable the dentist to care for a much larger clientele than he could otherwise do and render service to his patients that would entirely meet our present-day conception of preventive dentistry. Indeed, we have almost solved the question of control, if not cure, of the most prevalent and heretofore most intractable mouth condition—pyorrhea alveolaris—through the intelligent application of prophylactic measures, and in this field we believe the most brilliant results will be derived from the efforts of the dental hygienist in the way of preventive dentistry.

### PREVENTIVE DENTISTRY

The profession has long ago come to a realization of the hopelessness of fulfilling its mission through the means of reparative dentistry and is now concentrating its efforts in the direction of prevention. Varied efforts from various quarters are being made to solve the problem of caries causation with some hope of success. Research work in this field is encouraging, and we believe the most promising field of discovery in this line is in the direction of diet. The character of the saliva undoubtedly has an important bearing on the prevalence of caries, but when the much-discussed question of vitamines is better understood, it will very likely throw light on caries causation.

The greatest difficulty the profession has to deal with is lack of interest in research. The average dentist is so busily engaged in practice that he has little inclination in this direction, and for this the conditions under which he works are largely to blame.

Dentistry is a profession that makes enormous demands on the nervous energy and is becoming more difficult each year. The field is broadening and has become so large that it is fast developing by force of necessity into specialized branches and herein lies the hope for increased technical skill. The orthodontist has shown the way and when we have highly-skilled specialists in root canal technique, crown and bridge work, and other branches, we shall in all probability be able to handle the various phases of dentistry as successfully as the orthodontist handles his special phase.

It may appear to some that the writer has drifted far from his subject, but to sum up his impression of the present-day tendencies in dental practice he would say that the tendency is unmistakably in the direction of perfection of technical skill intelligently applied, with a full knowledge and understanding of clinical conditions evolved through scientific research and clinical experience, and toward rationalism in the practice of preventive dentistry as the only hope of properly fulfilling the humanitarian mission of dentistry as a branch, and a most important one, of the great healing art.

